# The Effect of the Forging Process on the Microstructure and Mechanical Properties of a New Low-Cost Ti-5Al-1.5Mo-1.8Fe Alloy

**DOI:** 10.3390/ma16145109

**Published:** 2023-07-20

**Authors:** Jinbao Hu, Yiqiang Mu, Qinsi Xu, Nan Yao, Shujun Li, Xiaofei Lei

**Affiliations:** 1 College of Civil Aviation, Shenyang Aerospace University, Shenyang 110136, China; hujinbao0831@163.com (J.H.); 20180053@sau.edu.cn (Y.M.);; 2 Institute of Metal Research, Chinese Academy of Sciences, Shenyang 110016, China

**Keywords:** a new low-cost titanium alloy, forging, microstructure, mechanical properties, fracture toughness

## Abstract

This paper presents results on the microstructure and mechanical properties of a new low-cost titanium alloy Ti-5Al-1.5Mo-1.8Fe after different forging processes. The β phase transformation temperature of this alloy was 950 °C. In this study, the forging temperatures were designed at 920 °C and 980 °C, and the deformation degree ranged from 20% to 60%, with an interval of 20%. This study investigated the impact of the equiaxed α phase and shape of the lamellar microstructure on the tensile characteristics and fracture toughness of an alloy. The research employed a microstructure analysis and static tensile testing to evaluate the effect of forging temperatures and degree of deformation on the microstructure features. The findings revealed that forging temperatures could modify the microstructure characteristics, and the degree of deformation also affected this microstructure. This study demonstrates that a bimodal structure with an equiaxed α phase can be utilized to balance high strength and high ductility, resulting in better overall mechanical properties.

## 1. Introduction

Titanium and its alloys are widely used in aerospace, the chemical industry, and load-bearing medical implants due to their exceptional mechanical, corrosive resistance, and biocompatible properties [1,2,3]. However, the high production cost of these titanium alloys has hindered their widespread use. Recently, researchers have focused on developing low-cost titanium alloys. Among them, Ti-Al-Mo-Fe systems have shown a high, dependable, and competitive mechanical property balance in the hot deformed, annealed, and thermally strengthened stages [4]. Moreover, Ti-Al-Mo-Fe alloy systems have been proven to be a promising alternative for developing complete β-Ti alloys with a low cost, high strength, and low elastic modulus, because a low-cost Mo-Fe master alloy can be used to stabilize the β phase at room temperature [5]. Compared to the majority of beta stable elements, alpha stabilizers are more plentiful and less expensive. Al can increase strength and thermal stability [6,7,8]. However, an Al content exceeding 6 wt% will reduce the plasticity due to a precipitate brittle Ti_3_Al phase [7,9]. Low-priced Mo-Fe master alloys have been used as alloying elements for their cost. Only a 10% weight of molybdenum (Mo) is required to stabilize the β phase at room temperature, making it a potent β-stabilizing element [10]. The addition of Mo reduces the elastic modulus and increases the strength of titanium alloys [11]. The addition of Mo to Ti-5Mn has successfully promoted the mechanical twinning of the alloys and improved their ductility [12]. Among the eutectoid β-stabilizing elements, Fe is the most commonly used substitute for isomorphous β-stabilizers due to its low cost and strong β-stabilizing capabilities [13,14,15]. Fe is a commonly used strengthening element. Rongjun Xu et al. reported that an addition of Fe to a Ti-Al-Mo-Fe alloy enhanced the room-temperature strength of the alloy and reduced its cost [7]. The addition of Fe to a Ti-Al system enhances the strength and lowers the liquidus temperature of the alloy, improving its thermo-mechanical properties, and Ti-Al-Fe alloy systems have also been effective at reducing costs [9]. However, an Fe content above 2 wt% will reduce the ductility. The elongation of low-cost Ti-5Al-3Mo-2Fe [7] alloys in the as-rolled and annealed state is 8.5%. The elongation of low-cost Ti-5Al-2.5Fe [9] alloys in the as-cast state is only 6%. Controlling the elemental content is critical to regulating an alloy’s properties.

The mechanical properties of alloys can be tailored by changing the morphology and distribution of the alpha phases and intermetallic compounds using thermomechanical treatments such as hot forging [9]. Forging is a common processing method for titanium alloys, and previous studies have demonstrated that deformation is an effective approach to achieving superior mechanical properties [16]. Ref. [17] states that the service life and reliability of Ti-6Al-4V alloy components can be effectively improved by designing suitable forging process parameters, including optimizing the forging temperatures while minimizing the scrap and energy consumption generated during the forging production process. Titanium components are forged below the β phase transition temperature to achieve the desired microstructure [18]. Forging plastic-forming processes, commonly used for producing Ti-6Al-4V alloys, can improve their mechanical properties. In Ti-Mo-Fe alloys, combining different plastic deformation mechanisms has been found to enhance the strength–ductility trade-off [19]. A high deformation rate during forging results in finer grains, and the primary strengthening mechanism employed during forging is grain refinement strengthening [17,20]. Lou et al. investigated the impact of different forging parameters on the phase transformation of a Ti-6Al-4V alloy during hot hammer forging [21]. Therefore, controlling the forging temperature is crucial when performing hot hammer forging plastic-forming processes, as it significantly influences the resulting forging properties [17]. Y. Chong et al. revealed that a bimodal microstructure with an equiaxed primary α had an excellent balance between its strength and ductility [22,23].

The material studied in this paper is Ti-5Al-1.5Mo-1.8Fe with an Al content of no more than 6% and an Fe content of no more than 2%. This composition was chosen to ensure that the material exhibited both a sufficient strength and a high ductility. The mechanical properties were investigated by examining the effects of different forging temperatures and deformation degrees. The purpose of this study was to research the influence that different forging temperatures and deformations have on the microstructural and mechanical properties of a new low-cost Ti-5Al-1.5Mo-1.8Fe alloy.

## 2. Material and Experimental Procedures

This study involved the use of commercially available grade 1 sponge titanium, as well as intermediate Al-Mo and Fe-Mo alloys, and Fe and TiO_2_ powder in the preparation of the alloy ingots. These materials were combined according to specific ratios and enclosed in a compacted alloy package, before being utilized as electrodes in a double vacuum consumable arc melting process. This approach was employed to achieve the efficient and cost-effective melting of the ingots. The chemical composition of the alloy used in this study was Ti-5Al-1.5Mo-1.8Fe (in wt.%) and the oxygen content was 0.070 (wt.%).

The Mo equivalent ([Mo]_eq_) [24], according to the following equation:

(1) $${\left[\mathrm{Mo}\right]}_{\mathrm{eq}}=1.0\left[\mathrm{Mo}\right]+2.9\left[\mathrm{Fe}\right]-1.0\left[Al\right]\left(\mathrm{wt}\%\right)$$

**Figure 1:**
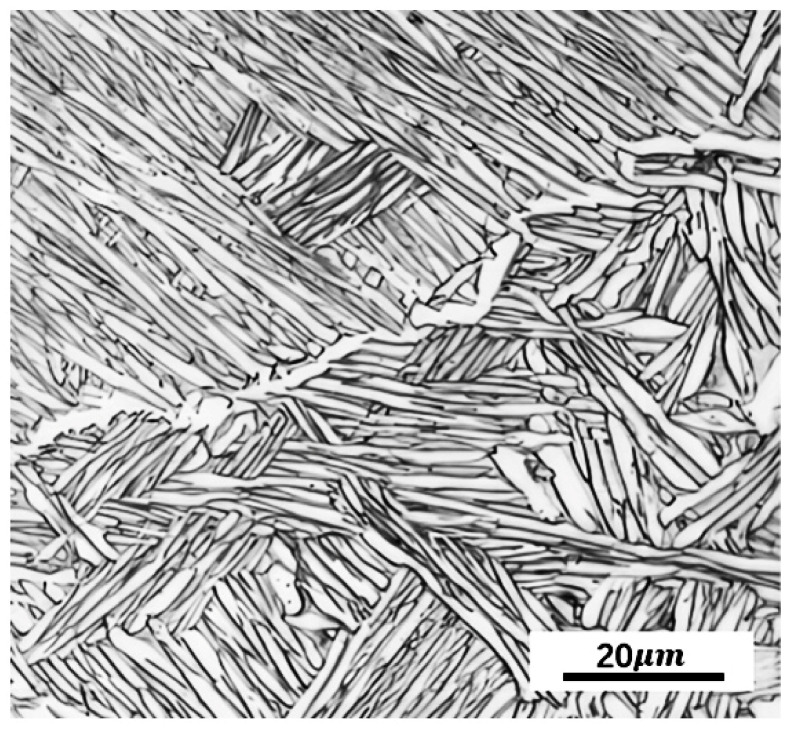
Optical micrograph of as-received Ti-5Al-1.5Mo-1.8Fe alloy.

Equation (1) states that the [Mo]_eq_ of Ti-5Al-1.5Mo-1.8Fe can be 1.76 wt%, which is an α + β two-phase titanium alloy [25]. As is shown in Figure 1, the Ti-5Al-1.5Mo-1.8Fe alloy had an initial microstructure with a widmanstätten morphology, where elongated α grains with a large aspect ratio were tightly packed within primary β grains.

Temperature is widely acknowledged as a critical factor that significantly impacts the performance in the fabrication of components [26,27,28]. The β phase transformation temperature was about 950 °C, which was measured using the metallographic method. This translated into two distinct forging techniques for the Ti-5Al-1.5Mo-1.8Fe alloy, namely α + β forging (T = T_β_ − 30 °C) and β forging (T > T_β_). Below the β transition temperature was the two-phase region, and above it was the single-phase region. The forging experiment plan is shown in Table 1, and the cooling method was air cooling.

**Figure 2:**
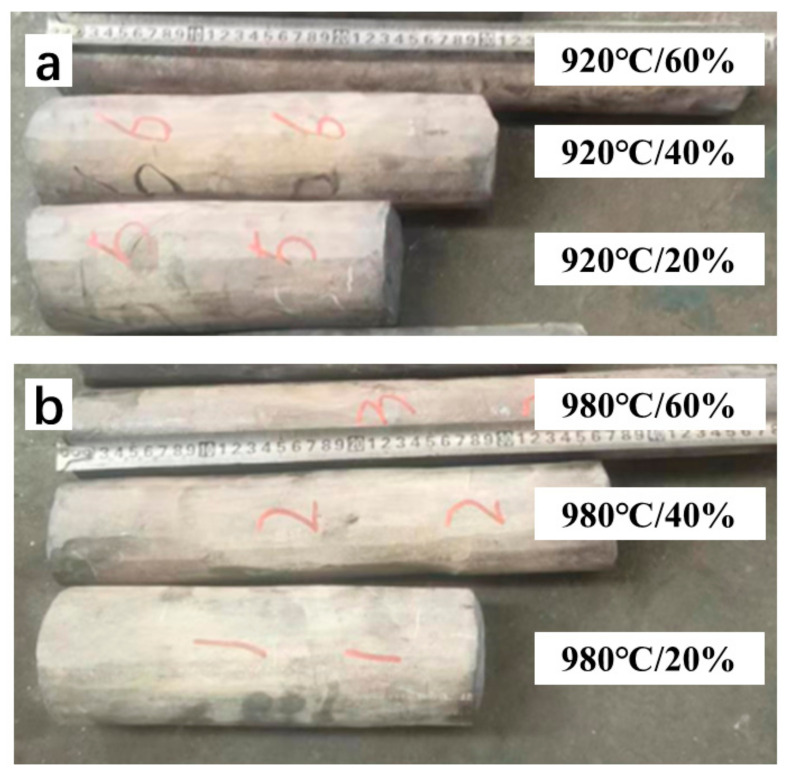
Overall shape of each deformation degree after forging, (**a**) different forging deformation of alloys at 920 °C, (**b**) different forging deformations of alloys at 980 °C.

Figure 2 shows the shape of the Ti-5Al-1.5Mo-1.8Fe alloy after forging, and the surface had not developed any external cracks.

**Figure 3:**
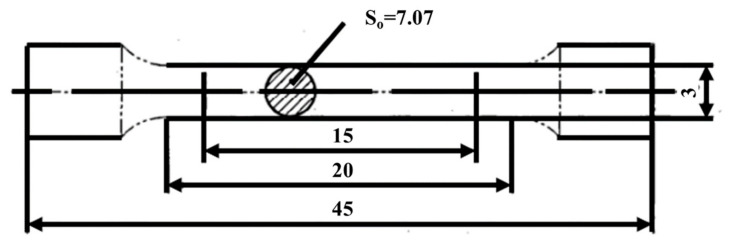
Schematic illustration of specimens used for the tensile tests. All dimensions are in mm.

The phase compositions of the studied alloys were identified using Cu-Kα X-ray diffractometry (XRD) with a diffractometer (Rigaku Smartlab equipment) operating at 45 kV and 200 mA in a 2θ range of 25–75°. The software MDI Jade 6 and OriginPro 2021 were used to process the XRD data. Samples for the microstructural evaluations were obtained from a central plane section and subjected to optical microscopy (OM) and scanning electron microscopy (SEM) analyses. Prior to these analyses, the specimen surfaces were wet ground using SiC papers of 150, 800, 1200, and 3000 grit and polished with SiO_2_ emulsion. To facilitate corrosion, the specimens were immersed in a solution containing 5 HF, 10 HNO_3_, and 85 H_2_O for 20 s. Tensile samples were obtained from each forged sample and subjected to room-temperature tensile testing. Before testing, all the samples were mechanically polished, cleaned, and dried. The tensile tests were conducted at room temperature using a SANS-CMT 5205 testing machine with a crosshead speed of 1mm/min. The shape of the tensile sample is shown in Figure 3. Each condition was evaluated by conducting tensile tests on two parallel specimens, and the ultimate tensile strength (UTS), yield strength (YS), elongation (EI), and reduction in area (RA) were determined from these tests. Subsequently, the plastic deformation and fracture behavior were analyzed using a TESCAN MIRA3 scanning electron microscope.

## 3. Results and Discussion

### 3.1. Effect of Processing on Microstructure

**Figure 4:**
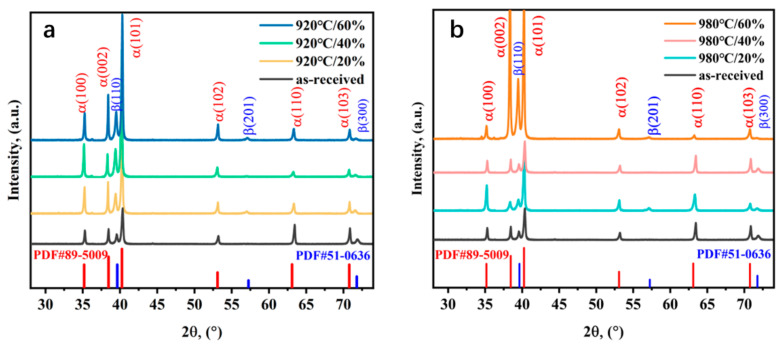
XRD patterns of Ti-5Al-1.5Mo-1.8Fe alloys. (**a**) Forging at 920 °C, (**b**) forging at 980 °C.

Figure 4 shows the XRD patterns of the Ti-5Al-1.5Mo-1.8Fe alloys before and after different degrees of the forging deformation process. The XRD analysis revealed the presence of body-centered cubic (bcc) β and hexagonal closed-packed (hcp) α phases in all of the studied samples. As the forging temperature of the alloy increased, the intensity of the peaks corresponding to the α(002), α(101), α(102), and β(110) phases substantially increased, as shown in Figure 4. As the forging deformation of the alloy increased, the intensity of the peaks corresponding to the α(100), α(002), α(101) and β(110) phases increased substantially, indicating an increase in the lattice density. The lower intensities of the identified α(101), α(110), and β(300) diffraction peaks after the forging deformation processing can be interpreted for the microstructural refinement during this postproduction process [29]. Comparing the different forging deformation of those samples, no distinct change in the peak positions was visible, indicating that the lattice constants were weakly affected by the amount of forging deformation.

**Figure 5:**
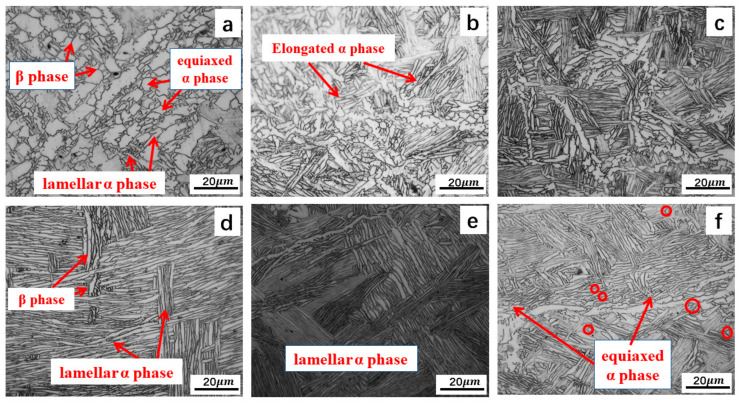
Microstructure under the different forging process: (**a**–**c**) forged at 920 °C with deformations of 20%, 40%, and 60%, respectively; (**d**–**f**) forged at 980 °C with deformations of 20%, 40%, and 60%, respectively.

The microstructures of the Ti-5Al-1.5Mo-1.8Fe alloys at different deformation temperatures and degrees are shown in Figure 5. The microstructures after forging at 920 °C are shown in Figure 5a–c, and the microstructures after forging at 980 °C are shown in Figure 5d–f. Among them, a and d correspond to the microstructures achieved with a forging deformation degree of 20%, b and e at 40%, and c and f at 60%.

The deformation temperature had a significant impact on the microstructure. The microstructure of the Ti-5Al-1.5Mo-1.8Fe alloy in Figure 5a consisted of equiaxed primary α, lamella α, and inter-crystalline β when forged below the β transition temperature. Increasing the forging temperature over the β transition temperature resulted in a decrease in the primary α content at the same deformation, as seen in Figure 5d. Deformation temperatures up to 980 °C led to a complete transformation of the equiaxed α into a lamellar structure. This lamellar structure could be observed (Figure 5d–f) when the deformation temperature was up to 980 °C. A widmanstätten microstructure formed after air cooling, characterized by coarse β grains with numerous α lamellar structures. During forging in the β single-phase region, the lack of pinning effects from the α grains led to an accelerated diffusion of the alloying elements within the β grain, resulting in rapid grain growth and deformation. The original β grains elongated in the deformation direction and the α phases were arranged in a lamellar structure within the grains [30,31].

The microstructure of a material is significantly impacted by forging deformation [32,33,34]. As this deformation increases, the degree of the alloy structure fragmentation also increases, leading to a smaller grain size, greater grain density, and increased mutual restraint between grains. The primary α grains were flattened, distorted, and broken-up with an increase in the deformation degree, and the part primary α phase recrystallized dynamically as the degree of deformation increased. As shown in Figure 5a, forging at low deformation rates below the β transition temperature provided enough time for recrystallization, leading to the formation of a larger primary α phase. Based on Figure 5b,c, it was observed that, when the deformation exceeded 40%, the temperature of the alloy surpassed the β transition point. This could result in the transformation of some equiaxed α grains into lamellar α grains and the creation of a basketweave structure in certain areas. It can be inferred that, during the forging process, a portion of the energy absorbed by the material was converted into internal energy of the plastic deformation. The diffusion pathway of this internal energy was dependent on the degree of forging deformation. As the deformation degree increased and the plastic deformation time was shortened, the internal energy could not diffuse to the surrounding medium and remained in the alloy material. Increases in both the distortion energy and dislocation density within the alloy were observed as the degree of deformation increased [35,36].

### 3.2. Effect of Parameters on Mechanical Property

**Figure 6:**
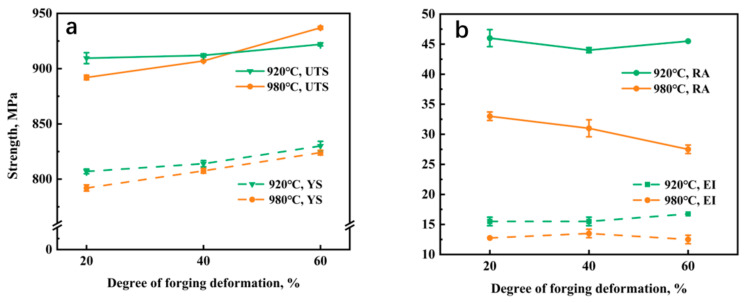
The room-temperature mechanical properties of Ti-5Al-1.5Mo-1.8Fe alloy after different deformation degree: (**a**) ultimate tensile strength (UTS) and the yield strength (YS), (**b**) reduction in area (RA) and elongation (EI).

Figure 6 illustrates the room-temperature mechanical properties of the Ti-5Al-1.5Mo-1.8Fe alloy under different forging temperatures and deformation degrees. The yield strength and ultimate tensile strength of the Ti-5Al-1.5Mo-1.8Fe alloy forged at 920 °C were found to be higher than those of the alloy forged at 980 °C at deformation degrees of 20% and 40%. However, forging the Ti-5Al-1.5Mo-1.8Fe alloy at 920 °C did weakly increase its strength, as evidenced by a yield strength increase from 807 MPa to 830 MPa and an ultimate tensile strength increase from 910 MPa to 922 MPa. In contrast, the strength of the alloy with a lamellar structure after forging at 980 °C continued to increase as the forging deformation increased, with a yield strength increase from 792 MPa to 824 MPa and an ultimate tensile strength increase from 892 MPa to 937 MPa. The reduction in area and elongation of the alloy forged at 920 °C were consistently higher than those of the alloy forged at 980 °C, with the maximum values of the elongation and reduction in area reaching 17% and 46%, respectively.

#### 3.2.1. Forging Temperature

The effect of the forging temperature on the mechanical properties is significant. The Ti-5Al-1.5Mo-1.8Fe alloy showed a better plasticity when forged at 920 °C, which was related to the presence of an equiaxed α phase. In the microstructure containing an equiaxed α phase, the mean-free-path of the α phase played a pivotal role in the tensile ductility of the alloy. The formation and growth of cavities were observed on interphase boundaries between the equiaxed α and transus β phases during the application of small tensile strain. The presence of primary α grains hindered the expansion of these cavities, with an increased primary α content resulting in a decrease in mean-free-path and a subsequent increase in cavity resistance. Consequently, the cavities encountered more barriers during growth, ultimately leading to an enhanced tensile deformation and high tensile ductility. In an alloy microstructure where the β matrix contains an equiaxed α phase, the mean free path of particles can significantly affect the tensile ductility of the alloy [37,38]. When the Ti-5Al-1.5Mo-1.8Fe alloy was forged above the phase transition temperature at 980 °C, the widmanstätten microstructure was formed. This microstructure is characterized by a regular arrangement of lamellar α phases within β grains. The α bundles share the same habit plane, and the slip can easily propagate and form a rapidly developing coarse slip band. Additionally, the presence of dislocation pile-up at the grain boundary α can lead to the premature formation and development of voids. These factors can ultimately weaken the resistance to crack initiation and reduce its plasticity.

**Figure 7:**
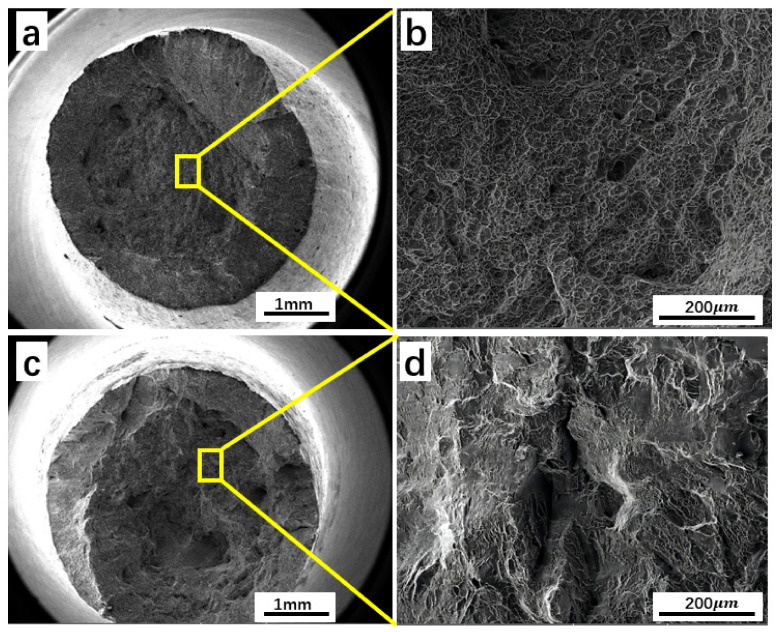
At a deformation of 20%, macroscopic fractography of two-phase region forging (**a**,**b**), macroscopic fractography of single-phase region forging (**c**,**d**).

Figure 7 shows the tensile fracture morphology of the alloy under different forging processes. The morphologies of the fracture surfaces forged below the β transition temperature at 920 °C are shown in Figure 7a,b. The areas where cracks propagate through the fibrous and shear lip regions are shown in Figure 7a. The macroscopic fracture surface of forging below the β transition temperature appears largely black, with visibility of the fiber region in the center and the cup-shaped shear lip on the outside. Figure 7b displays numerous dimples of varying sizes that correspond to the microscopic characteristics of a ductile fracture. The presence of these dimples indicates that the material possesses a good ductility, which is consistent with high cross-sectional shrinkage and elongation. Figure 7c,d show the microstructure of the tensile fracture of the lamellar structure after forging at a temperature above the β transition temperature (980 °C). When forging in the single-phase region, the microfracture surface exhibits features such as a few dimples, cleavage planes, and tear ridges, indicating induced embrittlement [39,40].

**Figure 8:**
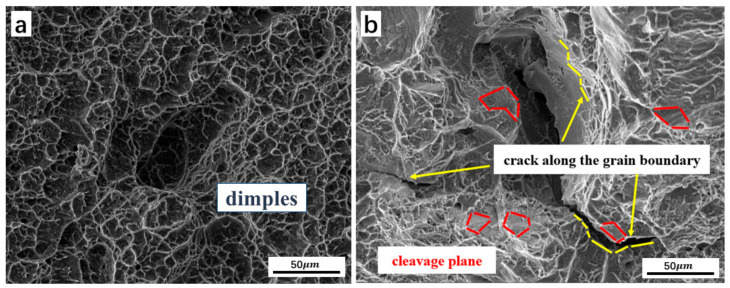
SEM micrographs of fracture surface, (**a**) dimples fracture, and (**b**) cleavage and crack propagating along the grain boundary.

Numerous dimples are observed in the two-phase region of the forged Ti-5Al-1.5Mo-1.8Fe alloy in Figure 8a, as revealed by its microstructure. This observation is consistent with the presence of ductile fracture on the surface. Ref. [4] also showed a similar conclusion, where the micro-level fracture surfaces had dimpled relief, indicating the ductile nature of the fracture. In contrast, cracks are visible in the single-phase region in Figure 8b, propagating along the grain boundaries. These findings confirm that the forging process led to a ductile fracture mechanism in the two-phase region and a quasi-dissociation fracture [40] mechanism in the single-phase region. Ref. [27] also showed a similar conclusion, where fewer and shallower dimples were observed. The fracture mode of the specimens exhibited a quasi-cleavage fracture, characterized by the gradual transformation of the fracture surface morphology into a cleavage plane with limited dimpling. The cleavage planes were linked by tearing edges, resulting in a fracture type that displayed both ductile and brittle fracture traits. The observed fracture morphology is consistent with the mechanical test results presented.

#### 3.2.2. Forging Deformation

At a forging temperature of 920 °C, more primary α grains showed a better plasticity. There was the presence of α colonies and a low degree of interweaving, which facilitates the passage of dislocations and leads to a diminished alloy strength [40]. The forging deformation exceeded 40%, and the plasticity of the alloy decreased because the reduction in the primary α. As the forging deformation increased, grain size refinement and an increased grain boundary area per unit volume prevented dislocation slip and more dislocation buildup occurred in the material, leading to an increased strength [27]. At a forging deformation degree of 60%, the ultimate tensile strength of the alloy forged at 980 °C was higher than that of the alloy forged at 920 °C. This can be explained by the fact that, when forging above the β transition temperature, a lamellar structure characterized by coarse β grains with numerous α lamellars was formed after air cooling, with grains elongated in the deformation direction and the α grains arranged in a lamellar structure within the grains. The resulting lamellar structure exhibited a relatively poor plastic deformation capacity, a phenomenon referred to as “β brittleness” [41].

**Figure 9:**
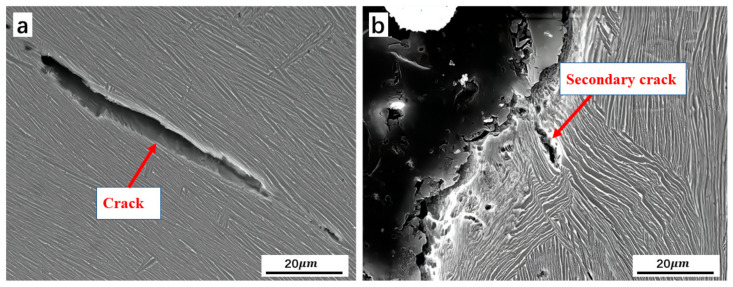
Crack-propagating path in lamellar microstructure (**a**) crack propagating along α/β interface, (**b**) secondary crack during crack propagating.

Increasing the degree of deformation in the Ti-5Al-1.5Mo-1.8Fe alloy could result in the development of a lamellar structure. Refs. [42,43,44] stated that alloys with a lamellar microstructure exhibit an improved strength due to modifications in the crack direction and the appearance of secondary crack branching. Figure 9a shows that modifying the crack path along the α/β phase boundary in a lamellar structure changes the crack propagation direction. In addition, Figure 9b demonstrates that crossing the boundaries between colonies may trigger the formation of secondary cracks, which require more energy [37]. Ref. [40] also showed a similar conclusion, where secondary cracks and a dissociation surface could be observed simultaneously, showing the characteristics of quasi-dissociation fracture.

## 4. Conclusions

(1) Forging the Ti-5Al-1.5Mo-1.8Fe alloy at 980 °C in the single-phase area resulted in a lamellar structure, while forging at 920 °C in the two-phase region generated a bimodal microstructure with equiaxed grains.
(2) The Ti-5Al-1.5Mo-1.8Fe alloy forged in the two-phase region had a superior plasticity, with a ductile fracture displaying many dimples. The alloy forged in the single-phase zone exhibited an inferior ductility, showing a crack fracture with numerous tear ridges, indicating that cracks propagated along the grain boundaries.
(3) The Ti-5Al-1.5Mo-1.8Fe alloy with the bimodal microstructure indicated a higher plastic deformation capacity compared to the lamellar structure. Although the strength of the lamellar structure increased with deformation, its ductility was compromised due to β-brittleness. The two-phase region forging achieved a balance between high strength and ductility, resulting in better overall mechanical properties.

## Figures and Tables

**Table 1:** Forging experiment scheme of Ti-5Al-1.5Mo-1.8Fe alloy at different forging process.

No.	Forging Types	Forging Temperature/°C	Forging Deformation/%
1	α + β	920	20
2			40
3			60
4	β	980	20
5			40
6			60

## Data Availability

The raw/processed data required to reproduce these findings cannot be shared at this time because the data also forms part of an ongoing study.
